# Intrapatient comparisons of efficacy in a single-arm trial of entrectinib in tumour-agnostic indications

**DOI:** 10.1016/j.esmoop.2021.100072

**Published:** 2021-03-04

**Authors:** M.G. Krebs, J.-Y. Blay, C. Le Tourneau, D. Hong, L. Veronese, M. Antoniou, I. Bennett

**Affiliations:** 1 Division of Cancer Sciences, Faculty of Biology, Medicine and Health, The University of Manchester, Manchester, UK; 2 Centre Léon Bérard, UNICANCER, Université Claude Bernard Lyon, Lyon, France; 3 Department of Drug Development and Innovation (D3i), Institut Curie, Paris-Saclay University, Paris & Saint-Cloud, France; 4 Department of Investigational Cancer Therapeutics, Division of Cancer Medicine, The University of Texas MD Anderson Cancer Center, Houston, USA; 5 F. Hoffmann-La Roche Ltd, Basel, Switzerland

**Keywords:** entrectinib, receptor protein-tyrosine kinases, neoplasms, comparative effectiveness research, treatment outcomes

## Abstract

**Background:**

Entrectinib is a tropomyosin receptor kinase inhibitor approved for the treatment of neurotrophic tyrosine receptor kinase (*NTRK*) fusion-positive solid tumours based on single-arm trials. Traditional randomised clinical trials in rare cancers are not feasible; we conducted an intrapatient analysis to evaluate the clinical benefit of entrectinib versus prior standard-of-care systemic therapies.

**Methods:**

Patients with locally advanced/metastatic *NTRK* fusion-positive tumours enrolled in the global phase II, single-arm STARTRK-2 trial were grouped according to prior systemic therapy and response. The key analysis used growth modulation index [GMI; ratio of progression-free survival (PFS) on entrectinib to time to discontinuation (TTD) on the most recent prior therapy]; ratio ≥1.3 indicated clinically meaningful efficacy. Additional analyses investigated TTD and objective response rate (ORR) for entrectinib and prior therapies.

**Results:**

Seventy-one patients were included; 51 received prior systemic therapy. In 38 patients who progressed on prior therapy, ORR was 60.5% (23/38) with entrectinib and 15.8% (6/38) with the most recent prior therapy. Median PFS [11.2 months; 95% confidence interval (CI) 6.7–not estimable] for entrectinib exceeded median TTD (2.9 months; 95% CI 2.0-4.9) for most recent prior therapy. From the intrapatient analysis of GMI, 65.8% had a ratio ≥1.3 and median GMI was 2.53. Consistent results were observed at more stringent GMI thresholds; 60.5% of patients had GMI ≥1.5 or ≥1.8 and 57.9% had GMI ≥2.0.

**Conclusions:**

ORR was high and PFS was longer on entrectinib versus TTD on prior therapy. Furthermore, 65.8% of patients experienced clinically meaningful benefit based on GMI. This intrapatient analysis demonstrates comparative effectiveness of entrectinib in a rare, heterogeneous adult population.

## Highlights

• Randomised trials are unfeasible for molecular targeted agents in rare indications because of low patient numbers.
• Intrapatient comparison with prior therapies can be used to evaluate relative treatment efficacy in rare tumours.
• Entrectinib is a potent tropomyosin receptor kinase (TRK) inhibitor with proven efficacy in neurotrophic tyrosine receptor kinase (*NTRK*) fusion-positive tumours from the global STARTRK-2 trial.
• Median progression-free survival on entrectinib was longer than time to discontinuation on prior therapy (11.2 months versus 2.9 months).
• About 61% of patients with prior therapy progression responded to entrectinib; 66% had growth modulation index ≥1.3 (clinically meaningful threshold).

## Introduction

Randomised clinical trials are the gold standard for assessing the clinical efficacy and safety of new drugs. However, comparative clinical trials are challenging in rare cancers, due to the limited number of patients who can be recruited. Evaluation of tumour-agnostic molecularly targeted agents (MTAs) poses further challenges due to the heterogeneity of tumour types and previous treatment regimens that patients may have received, which vary widely across treatment settings and geographies. Standard approaches to assessing response to therapy in clinical trials, that is, reduction in size of target lesions using RECIST, may also need to be reconsidered in this setting. For example, Le Tourneau and colleagues^1^ assessed tumour growth kinetics before and after treatment with MTAs and showed the value of this measure in addition to RECIST response categories. It was noted that a large proportion of patients discontinued MTA therapy as, despite a reduction in tumour growth rate while on treatment, RECIST response criteria were not met.^1^

Alternative efficacy endpoints to support antitumour effectiveness have been explored for evaluating signs of clinical benefit in small patient populations. An example is the growth modulation index (GMI), which is defined as the ratio of progression-free survival (PFS)/time to progression (TTP) on current therapy to PFS/TTP on the most recent prior therapy, within the same patient.^2^, ^3^, ^4^ This ratio can be used to determine whether current therapy is providing clinical benefit, and was originally proposed as a novel surrogate endpoint in the context of noncytotoxic drug trials, where a TTP endpoint is more appropriate than measuring tumour shrinkage.^2^ Considering that MTAs may generate significant clinical benefits aside from the tumour shrinkage [partial response (PR) or complete response (CR)] evaluated by RECIST, GMI has since been applied in early development settings to assess the benefit of targeted therapies selected by molecular profiling in patients with advanced refractory cancers.^4^, ^5^, ^6^, ^7^, ^8^ The benefit of using GMI for an intrapatient analysis is that patients act as their own controls, allowing the direct comparison of different treatments within the same patient over time, and this could be one approach to generate comparative efficacy data for a drug developed in single-arm trials.

Neurotrophic tyrosine receptor kinase (*NTRK*) gene fusions are rare; however, they can act as oncogenic drivers in a variety of cancer types,^9^ occurring in ~0.3% of all solid tumours.^10^ The tropomyosin receptor kinase (TRK) inhibitors entrectinib and larotrectinib have both received US Food and Drug Administration approval and European Medicines Agency approval, with a tumour-agnostic indication for *NTRK* fusion-positive cancers, based on single-arm trials (i.e. with no control arm).^11^^,^^12^ The objective of this analysis was to generate and analyse evidence for the comparative effectiveness of entrectinib, by exploring the role of intrapatient comparisons as an alternative to a traditional comparator arm.

## Material and methods

### Study design

Analyses used retrospectively collected data from the ongoing, phase II, open-label, multicentre, single-arm STARTRK-2 trial (NCT02568267) to generate intrapatient comparisons. The STARTRK-2 study design has previously been described.^11^ In brief, adult patients with metastatic/locally advanced *NTRK* fusion-positive solid tumours with measurable disease, and an Eastern Cooperative Oncology Group performance status ≤2, who had not received previous TRK targeted treatments (previous treatment with other cancer therapies was allowed) were enrolled. Central nervous system (CNS) metastases were permitted. *NTRK* gene fusion status was tested by local molecular profiling [fluorescence *in situ* hybridisation tests, quantitative polymerase chain reaction, or DNA- or RNA-based next-generation sequencing (NGS)] or central RNA-based NGS (Trailblaze Pharos). Patients enrolled by local testing were required to provide tumour tissue (unless a biopsy was medically contraindicated) for independent central NGS testing. Patients received continuous 600 mg once daily dosing of entrectinib. Tumour assessments, performed at the end of week 4 and every 8 weeks thereafter, were evaluated by blinded independent central review (BICR) using RECIST version 1.1. STARTRK-2 was conducted in accordance with the principles of the Declaration of Helsinki and Good Clinical Practice Guidelines. The protocol was approved by the relevant institutional review boards and/or ethics committees. Written informed consent was obtained from all patients.

### Intrapatient analysis cohorts

Patients were considered in three cohorts based on prior systemic therapy in the metastatic setting and presence/absence of documented progression. The ‘documented progression on prior therapy’ cohort comprised patients who had received at least one systemic therapy for metastatic disease prior to commencing entrectinib and clear documentation of progressive disease (PD) on the most recent prior therapy, as captured in electronic case report forms. The ‘no documented progression on prior therapy’ cohort comprised patients who had received at least one systemic therapy for metastatic disease prior to commencing entrectinib and had no documentation of PD on the most recent prior therapy. This cohort included patients who stopped prior therapy due to toxicity, completion of the course, or other reasons. The ‘no prior therapy cohort’ comprised patients who had received no prior systemic therapy for metastatic disease before starting entrectinib, though they may have received prior (neo)adjuvant therapy.

### Study endpoints and analyses

For entrectinib, time to discontinuation (TTD) for any reason was defined as time from start of entrectinib until end of entrectinib therapy, and PFS was defined as the time from the first dose of entrectinib to first documentation of radiographic disease progression or death due to any cause, whichever occurred first. For prior therapies, TTD was defined as time from the start of the most recent prior therapy until the end of the most recent prior therapy. If the start or end date was missing, a conservative imputation rule was applied; missing start dates of prior therapy were imputed as earliest possible date (i.e. 1 January or first day of the month), and missing end dates of prior therapy were imputed as latest possible date (i.e. 31 December, end of month, or start of entrectinib). For prior therapies, available data on assessment methods and dates were too limited to reliably define a TTP outcome, motivating the use of TTD. Patients receiving ongoing entrectinib therapy were censored for TTD; patients who had not progressed/died were censored for PFS. For entrectinib, responses and PFS were assessed by BICR using RECIST version 1.1. For prior therapies, response was assessed by the treating physician and recorded on the electronic case report form. Objective response rate (ORR) was defined as the proportion of patients achieving a CR or PR.

The key analysis compared the efficacy of entrectinib with that of prior systemic therapy using GMI in the documented progression on prior therapy group. GMI was defined as the ratio of PFS on entrectinib to TTD on the most recent prior therapy; these endpoints (PFS and TTD) were selected as the best indicators of drug efficacy based on available data for entrectinib and prior therapy, respectively. To assess the relevance of TTD as a measure of progression for prior therapies, the TTD and PFS of entrectinib were compared. A GMI ratio of ≥1.3 was set as the threshold to indicate a clinically meaningful benefit, based on previously described GMI cut-offs.^2^, ^3^, ^4^, ^5^, ^6^, ^7^ Additional analyses explored TTD and ORR for entrectinib and prior systemic therapy.

Kaplan–Meier methodology was used to explore median TTD on entrectinib or most recent prior systemic therapy as well as median PFS on entrectinib in the cohort with documented progression on prior therapy. A Kaplan–Meier analysis of GMI taking censoring into account was also performed. Time-to-event analysis used Kaplan–Meier methods as implemented using R statistical software. TTD and ORR were further investigated for individual patients in all three cohorts for entrectinib and for the most recent prior systemic therapy in the prior systemic therapy cohorts.

## Results

### Patients

**Figure 1:**
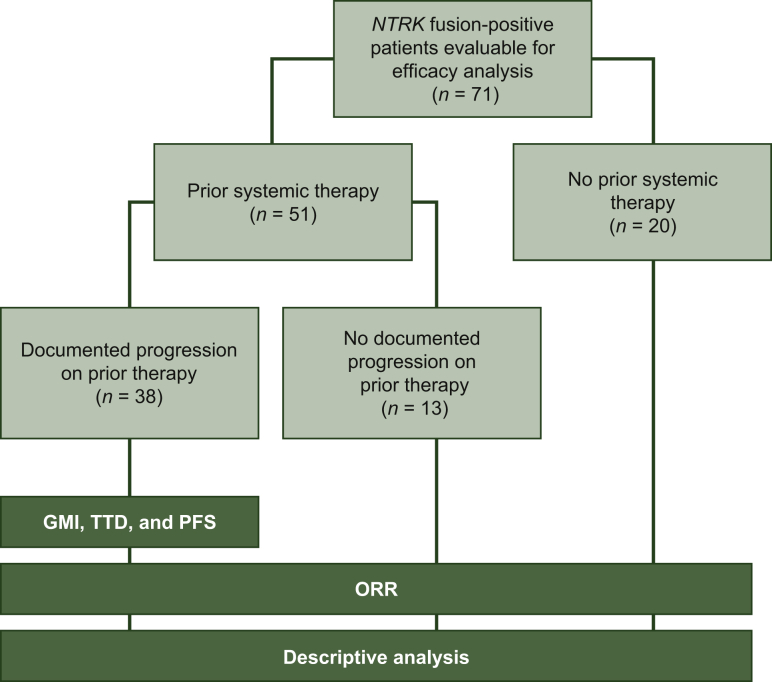
Disposition of patients from the STARTRK-2 trial according to prior systemic therapy and documented progression. Enrolled patients were grouped into three cohorts: documented progression on prior therapy, no documented progression on prior therapy, and no prior systemic therapy. Analyses performed and the cohorts included are shown in the dark green boxes. Documented progression included recorded reason for discontinuation of primary resistance/no response to therapy (*n* = 13), progressive disease (response followed by relapse) (*n* = 14), or other reason combined with a date for progression (*n* = 11). GMI, growth modulation index; *NTRK*, neurotrophic tyrosine receptor kinase; ORR, overall response rate; PFS, progression-free survival; TTD, time to discontinuation.

Seventy-one patients with efficacy-evaluable *NTRK* fusion-positive disease enrolled into STARTRK-2 up to 30 April 2018 (data cut-off 31 October 2018) were included in the analysis (GD Demetri et al., unpublished data).^13^ Overall, 51 patients had received systemic therapy prior to commencing entrectinib, of whom 38 had documented progression and 13 had no documented progression on the most recent prior systemic therapy (Figure 1); 20 patients had not received prior systemic therapy. Baseline characteristics are summarised in Table 1.

**Table 1:** Baseline characteristics of patient cohorts, according to prior therapy and documented progression

Characteristic	Documented progression on prior therapy (*n* = 38)	No documented progression on prior therapy (*n* = 13)	No prior therapy (*n* = 20)	Total (*N* = 71)
Type of cancer, *n* (%)				
Sarcoma	9 (23.7)	4 (30.8)	3 (15.0)	16 (22.5)
Non-small-cell lung cancer	6 (15.8)	3 (23.1)	3 (15.0)	12 (16.9)
MASC	5 (13.2)	1 (7.7)	6 (30.0)	12 (16.9)
Thyroid	4 (10.5)	1 (7.7)	2 (10.0)	7 (9.9)
Breast	1 (2.6)	2 (15.4)	3 (15.0)	6 (8.5)
Colorectal cancer	5 (13.2)	0	1 (5.0)	6 (8.5)
Pancreatic	1 (2.6)	1 (7.7)	1 (5.0)	3 (4.2)
Neuroendocrine	4 (10.5)	0	0	4 (5.6)
Gynaecological^a^	2 (5.3)	0	0	2 (2.8)
Cholangiocarcinoma	1 (2.6)	0	0	1 (1.4)
Upper gastrointestinal tract	0	0	1 (5.0)	1 (1.4)
Neuroblastoma^b^	0	1 (7.7)	0	1 (1.4)
CNS metastases present at baseline^c^	10 (26.3)	4 (30.8)	4 (20.0)	18 (25.4)
Fusion gene				
*NTRK1*	17 (44.7)	5 (38.5)	6 (30.0)	28 (39.4)
*NTRK2*	1 (2.6)	1 (7.7)	0	2 (2.8)
*NTRK3*	20 (52.6)	7 (53.8)	14 (70.0)	41 (57.8)
Fusion partner				
*ETV6*	17 (44.7)	4 (30.8)	12 (60.0)	33 (46.5)
*TPM3*	8 (21.1)	0	2 (10.0)	10 (14.1)
*TPR*	3 (7.9)	1 (7.7)	1 (5.0)	5 (7.0)
Other	10 (26.3)	8 (61.5)	5 (25.0)	23 (32.4)
Prior radiotherapy	22 (57.9)	9 (69.2)	15 (75.0)	46 (64.8)
Prior surgery	28 (73.7)	11 (84.6)	20 (100.0)	59 (83.1)

Characteristic	Documented progression on prior therapy (*n* = 38)	No documented progression on prior therapy (*n* = 13)	No prior therapy NA	Total (*N* = 51)
Lines of previous systemic therapy			NA	
1	17 (44.7)	4 (30.8)		21 (41.2)
2	16 (42.1)	4 (30.8)		20 (39.2)
≥3	5 (13.2)	5 (38.5)		10 (19.6)
Type of systemic therapy prior to entrectinib^d^			NA	
Chemotherapy	25 (65.8)	9 (69.2)		34 (66.7)
Targeted therapy	8 (21.1)	4 (30.0)		12 (23.5)
Immunotherapy^e^	7 (18.4)	0		7 (13.7)
Monoclonal antibody^e^	4 (10.5)	1 (10.0)		5 (9.8)
Hormone therapy	0	2 (15.4)		2 (3.9)
Best response to most recent line of therapy			NA	
CR	1 (2.6)	0		1 (2.0)
PR	5 (13.2)	1 (7.7)		6 (11.8)
SD	9 (23.7)	6 (46.2)		15 (29.4)
PD	15 (39.5)	0		15 (29.4)
Non-CR/non-PD	0	1 (7.7)		1 (2.0)
Not evaluable	1 (2.6)	2 (15.4)		3 (5.9)
Unknown	7 (18.4)	3 (23.1)		10 (19.6)

CNS, central nervous system; CR, complete response; MASC, mammary analogue secretory carcinoma; NA, not applicable; *NTRK*, neurotrophic tyrosine receptor kinase; PD, progressive disease; PR, partial response; SD, stable disease.

a Ovarian adenocarcinoma, *n* = 1; endometrial carcinoma, *n* = 1.

b One patient with neuroblastoma presented with a *SCAPER-NTRK3* fusion.

c CNS metastases at baseline as assessed by investigator.

d Therapy could be alone or a combination of chemotherapy with chemotherapy as maintenance; chemotherapy with hormone therapy; chemotherapy with monoclonal antibody; chemotherapy with targeted therapy; hormone therapy with targeted therapy; immunotherapy with targeted therapy.

e Immunotherapy included atezolizumab, avelumab, nivolumab, and pembrolizumab. Monoclonal antibody therapy included bevacizumab, cetuximab, olaratumab, panitumumab, and ramucirumab. See Supplementary Table S1, available at https://doi.org/10.1016/j.esmoop.2021.100072 for further details.

The most frequent tumour types were sarcoma (16/71, 22.5%), non-small-cell lung cancer (12/71, 16.9%), mammary analogue secretory carcinoma (12/71, 16.9%), and thyroid cancer (7/71, 9.9%). Among 51 patients who had received prior systemic therapy, 21 (41.2%) received one line, 20 (39.2%) received two lines, and 10 (19.6%) received three or more lines of therapy. The most recent prior therapy for the majority of patients was chemotherapy (34/51, 66.7%) either alone or in combination with other agents. However, treatment regimens varied greatly within and between tumour types (Supplementary Table S1, available at https://doi.org/10.1016/j.esmoop.2021.100072).

### Efficacy in all patient cohorts

The best overall response to therapy is summarised for all patients in Supplementary Figure S1, available at https://doi.org/10.1016/j.esmoop.2021.100072. The ORR for entrectinib was 60.5% (23/38; all PR) in patients with documented progression on prior therapy, 46.2% (6/13; all PR) in patients with no documented progression on prior therapy, and 80% (16/20; 5 CR and 11 PR) in patients with no prior therapy. The ORR for most recent prior systemic therapies was 15.8% (6/38; one CR and five PR) in patients with documented progression on prior therapy and 7.7% (1/13; PR) in patients with no documented progression on prior therapy.

Among 23 patients with documented progression on prior therapy who responded to entrectinib, 10 (43.5%) had never achieved a better response than PD on prior therapy. Among six patients with no documented progression on prior therapy who responded to entrectinib, four (66.7%) had experienced a best response of stable disease on prior therapy. Among 51 patients who had received prior systemic therapy, most patients (6/7, 85.7%) who had responded to most recent prior systemic therapy also responded to entrectinib.

### TTD and PFS in all patient cohorts

**Figure 2:**
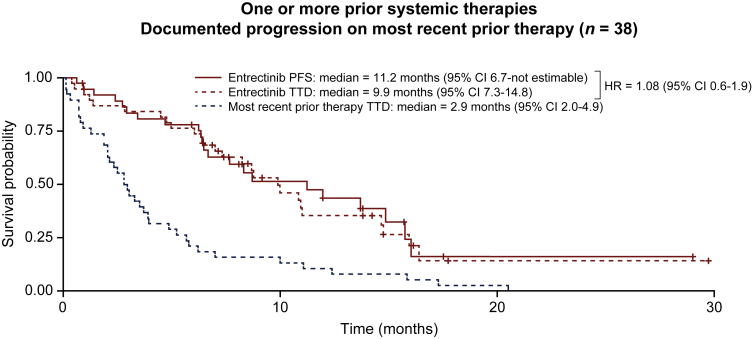
Kaplan–Meier curves of TTD on entrectinib versus the most recent prior systemic therapy and PFS with entrectinib in patients with documented progression on the most recent prior therapy (*n* = 38). Crosses indicate the patient has been censored. CI, confidence interval; HR, hazard ratio; PFS, progression-free survival; TTD, time to discontinuation.

**Figure 3:**
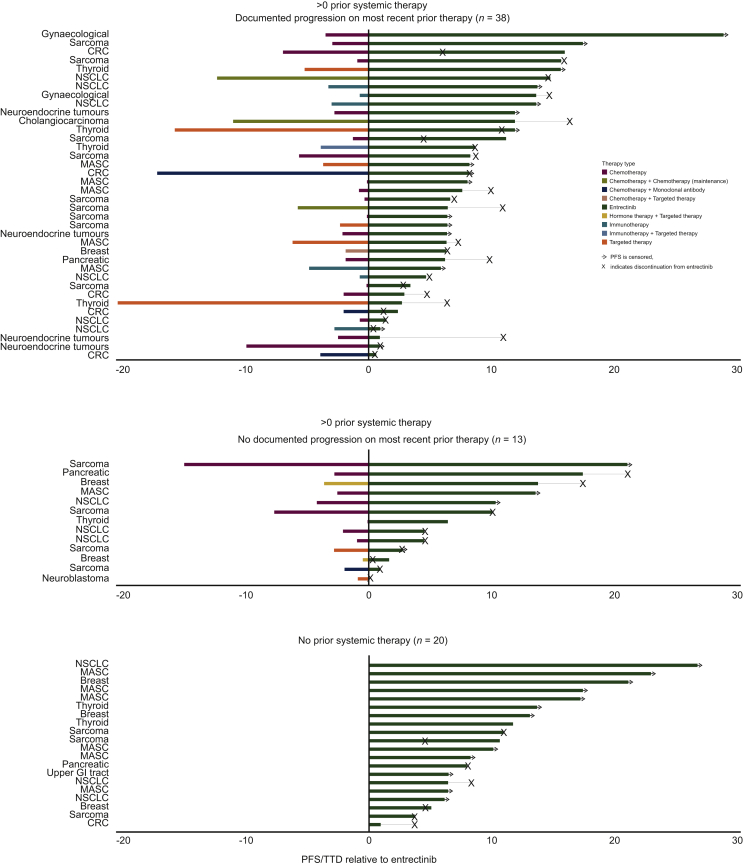
PFS on entrectinib and TTD on the most recent prior therapy in all patients. For entrectinib, PFS was defined as the time from the first dose of entrectinib to first documentation of radiographic disease progression or death due to any cause, whichever occurred first. For prior therapies, TTD was defined as time from the start of most recent prior therapy until the end of most recent prior therapy. Missing start and end date days were imputed via a conservative rule. Patients with ongoing entrectinib therapy are censored. CRC, colorectal cancer; GI, gastrointestinal; MASC, mammary analogue secretory carcinoma; NSCLC, non-small-cell lung cancer; PFS, progression-free survival; TTD, time to discontinuation.

Kaplan–Meier survival analysis in patients with documented progression on prior therapy and in all patients with prior therapy is shown in Figure 2. The curves for PFS and TTD on entrectinib were similar [hazard ratio of PFS to TTD, 1.08; 95% confidence interval (CI) 0.6-1.9], with median PFS on entrectinib of 11.2 months (95% CI 6.7–not estimable) and a median TTD on entrectinib of 9.9 months (95% CI 7.3-14.8). Consistency between TTD and PFS on entrectinib was observed within individual patients and for each cohort (Figure 3). Both PFS and TTD on entrectinib were longer than TTD on most recent prior therapy, which had a median of 2.9 months (95% CI 2.0-4.9; Figure 2).

### GMI in patients with PD on prior systemic therapy

**Figure 4:**
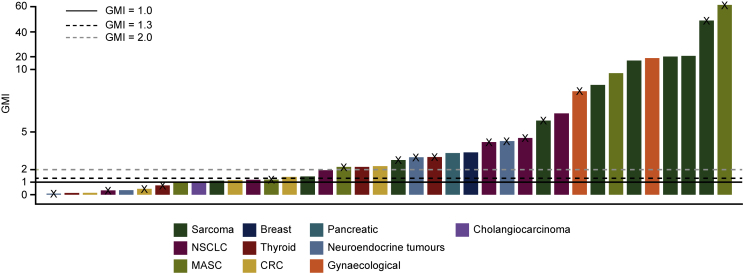
GMI for individual patients with documented progression on the most recent prior therapy. The GMI is derived from the ratio of PFS on entrectinib to the TTD on the most recent prior therapy. The dashed lines indicate a GMI ratio of 1.3 (threshold for clinically meaningful benefit; black) and 2.0 (grey). The black solid line indicates a ratio of 1.0. Crosses indicate that the patient's PFS is censored, as entrectinib treatment is ongoing. CRC, colorectal cancer; GMI, growth modulation index; MASC, mammary analogue secretory carcinoma; NSCLC, non-small-cell lung cancer; PFS, progression-free survival; TTD, time to discontinuation.

Individual GMI ratios for entrectinib versus the most recent prior therapy are presented in Figure 4; median GMI was 2.53 (range 0.09-61.5). Twenty-five patients (65.8%) had a GMI ≥1.3, indicating a clinically meaningful benefit with entrectinib; among these patients, 17 had a PR, 4 had stable disease, 1 had PD, 1 had non-CR/non-PD, and 2 were not evaluable.

Overall, four of seven patients (57.1%) with a GMI <1.0 on entrectinib were censored for PFS. Varying the GMI threshold for clinically meaningful benefit with entrectinib to ≥1.5, ≥1.8, and ≥2.0 led to consistent results, with 23 (60.5%), 23 (60.5%), and 22 (57.9%) patients meeting these thresholds, respectively.

In a Kaplan–Meier analysis taking censoring into account in these patients, median GMI was 6.5 (95% CI 2.3-20.3), and the probabilities of achieving GMIs of ≥1.3, ≥1.5, ≥1.8, and ≥2.0 were 0.77, 0.71, 0.71, and 0.68, respectively (Supplementary Figure S2, available at https://doi.org/10.1016/j.esmoop.2021.100072).

## Discussion

We report the use of an intrapatient analysis to overcome the challenges of investigating comparative efficacy of entrectinib in rare *NTRK* fusion-positive solid tumours through a traditional randomised clinical trial. GMI analysis of the ratio of PFS on entrectinib to TTD on most recent prior systemic therapy, using a GMI threshold of ≥1.3 for a clinically meaningful response, showed clinical benefit with entrectinib in 65.8% of patients with documented progression on prior therapy (i.e. not including discontinuations due to other causes). Median PFS (11.2 months) on entrectinib was also longer than median TTD (2.9 months) on most recent prior therapy. Several patients were still receiving entrectinib treatment and had their PFS or TTD censored; therefore, there is scope for further improvements in GMI and TTD after longer follow-up as patients experience progression or discontinue treatment.

Although the GMI (or PFS ratio) is not yet a validated efficacy endpoint due to an uncertain correlation between the two PFS values,^14^ it is clinically relevant for patients as they usually perceive a new treatment as effective when they experience benefit for longer period than on the previous line of therapy. Consequently, GMI is increasingly being used to facilitate assessment of MTAs in cancer therapy and has demonstrated the benefit of MTAs, in the form of prolonged PFS, compared with most recent conventional therapy.^4^, ^5^, ^6^, ^7^ However, while the duration of benefit on entrectinib is important, so too is the depth of benefit. Although GMI does not consider the impact of treatment on patient symptoms and quality of life, a phase II clinical trial reported strong correlation between a GMI >1.33 and improved response to treatment, longer median overall survival, and PFS, compared with a GMI ≤1.33.^15^ Most patients (*n* = 17; 68.0%) in our study with a GMI ≥1.3 were responders to entrectinib, but there were also six patients classed as nonresponders and two patients who died before being evaluated. This clinical benefit in nonresponders may not have been captured using traditional trial endpoints, albeit impact on patient symptoms and quality of life are not reported.

All previous GMI studies utilised the ratio of 1.3 or 1.33 to demonstrate clinically meaningful benefit, as proposed by Von Hoff.^2^^,^^4^, ^5^, ^6^, ^7^ A ratio of ≥1.3 or ≥1.33 rather than >1.0 was originally chosen to minimise false-positive fluctuations and limit overestimation of treatment effect. However, a recent analysis of patients enrolled into successive early-phase clinical trials found that 25% of patients had a TTP ratio >1.3 in the absence of an overall treatment effect.^14^ This finding led the authors to suggest that higher ratios (>1.8), or proportion of patients with ratio >1.3 exceeding 25%, may be appropriate for studies of agents with a known molecular target. In our analysis of entrectinib, 65.8% of patients (25/38) achieved a GMI ≥1.3, and 60.5% (23/38) achieved a GMI ≥1.8, with five patients below these thresholds having a censored PFS. This suggests a clinically meaningful benefit even when more stringent criteria are applied. Our GMI results are further supported by prolonged PFS on entrectinib compared with TTD on the most recent prior therapy, despite PFS being expected to decrease with successive lines of therapy.^16^

A recent intrapatient analysis compared larotrectinib with prior lines of therapy in patients with *NTRK* fusion-positive cancers (*n* = 72) using the GMI method.^17^ GMI was calculated as the ratio of PFS on larotrectinib to TTP on the most recent prior line of therapy; TTP was defined as the time from start of the last prior therapy to radiological or clinical progression or treatment failure. In the overall cohort (including 21 paediatric and 51 adult patients), median GMI was 2.68 (6.46 by Kaplan–Meier estimates) and 47/72 (65.3%) patients achieved a GMI ratio ≥1.33 on larotrectinib. Among patients with metastatic disease (*n* = 53), median PFS was 19.3 months, median GMI was 2.87, and 66.0% had a GMI ≥1.33. Importantly, the larotrectinib analysis included any patient with prior therapy; 24/72 (33.3%) patients had not progressed on prior therapy but had discontinued treatment due to stable disease, an adverse event, or a patient and/or physician decision. Some of these patients may thus have discontinued prior therapy prematurely in order to receive larotrectinib, resulting in an artificially low TTP on prior therapy and thus an artificially high GMI. By contrast, our analysis took a more conservative approach and focused solely on patients with documented progression on prior therapy, that is, those who discontinued prior therapy due to lack of efficacy, a cohort that provides the best means to directly assess the efficacy of entrectinib. It is also interesting to note differences in the patient populations between the two analyses. CNS metastases are associated with poor outcome^18^ and were reported in 26.3% of patients in our analysis but not reported in the larotrectinib analysis, though the larotrectinib pooled efficacy analysis reports only 8% of patients with CNS metastases.^19^ In addition, while our analysis included adult patients only, the larotrectinib analysis included 21 patients (29.2%) aged <18 years.^17^ Overall, our GMI analysis in adults supports the finding of clinically meaningful benefit of TRK inhibitors in patients with *NTRK* fusion-positive cancer, in patients who responded poorly to prior therapy.

Kaplan–Meier analyses showed that TTD on entrectinib and PFS by BICR on entrectinib were similar and both were longer than TTD on the most recent prior systemic therapy. Although PFS by BICR on entrectinib was available based on clinical trial data, the best available measure for prior treatment was TTD, which was used as a surrogate for PFS and may therefore overestimate PFS if patients continue on treatment beyond progression. Across cohorts, 46%-80% of patients responded to entrectinib compared with 8%-16% on the most recent prior systemic therapy. This, along with inclusion of patients with 12 different types of *NTRK* fusion-positive tumours, who received a range of different prior systemic treatments, suggests that the trial population was not a heavily selected population predetermined to be good responders. Furthermore, it highlights the challenges of clinical trials of tumour-agnostic therapy across multiple tumour types, the paucity of information around different natural histories of *NTRK* fusion-positive tumours, and uncertainty surrounding *NTRK* fusions as a prognostic marker, that is, expected survival of patients (compared with non-*NTRK* fusion-positive tumours) regardless of treatment received.

A recent study from the Memorial Sloan Kettering Cancer Center identified 76 patients with *NTRK* gene fusions from a cohort of >26 000 patients, to investigate genomic characteristics, therapies, and outcomes.^20^ Patients with advanced/recurrent disease (*n* = 51) with 15 different cancers underwent treatment with a range of chemotherapies, immunotherapies, and/or TRK inhibitors. ORR was 64.7% for targeted TRK inhibition, 62.5% for chemotherapy-containing regimens, and 11.1% for immunotherapy, although treatment sequence was not specified. ORR to all first-line therapies, excluding TRK inhibitors, was 46.7%. Treatment comparisons from the Memorial Sloan Kettering Cancer Center study are confounded by heterogeneity of tumour types between treatment groups, lack of information regarding baseline characteristics and prior treatment history, and lengthy overall survival (median 19.8 years) in this cohort. With the move towards personalised medicines, efficacy-evaluable populations harbouring specific genetic alterations are vastly reduced versus historical clinical trial populations. Alternative approaches for comparative efficacy such as GMI and collation of real-world evidence on clinical outcomes, that overcome these challenges, can be expected to play a more prominent role in future drug development. Indeed, health technology assessment bodies are beginning to recognise the value of such analyses as part of the evidence package for evaluation of targeted therapies in rare indications. In particular, intrapatient analyses can be available in a more timely manner than follow-on real-world studies, thus allowing for an early evaluation of comparative effectiveness^21^; by using patients as their own control, intrapatient analyses also eliminate between-patient variability.

Limitations of our analysis include the censoring of entrectinib data points due to ongoing treatment or treatment benefit, which may have resulted in a conservative estimate of the difference between entrectinib and prior systemic therapy. Our analysis comprises a small number of clinical trial patients who may not be representative of real-world patients with *NTRK* fusion-positive cancer. Although information on prior therapy was part of the study data collection, and underwent monitoring and source verification, a number of responses to prior therapy were unknown (overall, 21/100 documented responses out of all prior therapies received across 51 patients; 10/51 most recent prior therapy responses). For GMI analyses, it was assumed that tumour growth kinetics were linear over time, that is, the same at diagnosis, for prior therapies and at time of entrectinib treatment; however, tumour models suggest it may be exponential or logarithmic.^22^ The timing of tumour assessment was controlled for entrectinib but not for prior therapy; this may have impacted the date of progression and the GMI result. Similarly, RECIST was used to assess entrectinib response, but may not have been used for prior therapies. Although analyses used PFS by BICR for entrectinib, TTD on prior therapy was based on investigator assessment.

## Conclusions

We investigated intrapatient comparisons of response rates, PFS, TTD, and GMI on entrectinib and prior therapy to investigate comparative efficacy in patients with *NTRK* fusion-positive, locally advanced/metastatic solid cancers. Among patients who had progressed on the most recent prior therapy, 60.5% responded to entrectinib and 65.8% had a GMI ratio ≥1.3 (the clinically meaningful threshold). Although GMI has its limitations, the greater PFS on entrectinib versus TTD on prior therapy from our intrapatient analysis is strengthened by the high response rates on entrectinib compared with the most recent prior therapy. For future single-arm trials of tumour-agnostic agents, we recommend that intrapatient comparisons could be preplanned analyses and efforts should thus be placed on prospective discussions with regulatory authorities and collection of detailed prior therapy data and responses. Together, these results show the value and feasibility of using an intrapatient analysis to assess comparative effectiveness of tumour-agnostic MTAs in a heterogeneous patient population.
