# Supplementary material for: Intrapatient comparisons of efficacy in a single-arm trial of entrectinib in tumour-agnostic indications
Source: ESMO Open. 2021 Mar 4;6(2):100072. doi: 10.1016/j.esmoop.2021.100072 (PMC8103537; doi:10.1016/j.esmoop.2021.100072)
Supplement: Supplementary Material [file mmc1.docx]

**Supplementary Materials**

**Supplementary Table S1. Summary of prior systemic treatment** **according to tumour type in patients with at least one systemic prior therapy (n=51).**

| Tumour type | Treatment category | Treatment drug (number of patients) |
| --- | --- | --- |
| Sarcoma | Chemotherapy | Doxorubicin (n=3) Gemcitabine (n=2) Gemcitabine + docetaxel (n=3) Doxorubicin + ifosfamide (n=4) Gemcitabine + docetaxel + doxorubicin (n=1)  Ifosfamide (n=1) Ifosfamide + pegylated liposomal doxorubicin (n=1)  Temozolomide (n=1)  Doxorubicin + gemcitabine (n=1) |
|  | Chemotherapy + monoclonal antibody | Doxorubicin + olaratumab (n=1) |
|  | Chemotherapy + targeted therapy | Gemcitabine + docetaxel + pazopanib (n=1) |
|  | Hormone therapy | Anastrozole (n=1) |
|  | Targeted therapy | Pazopanib (n=1) Sunitinib (n=1) |
| NSCLC | Chemotherapy | Carboplatin + paclitaxel (n=2) Carboplatin + pemetrexed (n=4) Gemcitabine + carboplatin (n=1)  Paclitaxel albumin (n=1) |
|  | Chemotherapy + chemotherapy (maintenance) | Carboplatin + pemetrexed disodium + pemetrexed (maintenance) (n=1) |
|  | Chemotherapy + monoclonal antibody | Docetaxel + ramucirumab (n=1) Paclitaxel albumin + ramucirumab (n=1) Pemetrexed + carboplatin + bevacizumab (n=1) |
|  | Immunotherapy | Pembrolizumab (n=3) Nivolumab (n=2) Atezolizumab (n=1) |
| MASC | Chemotherapy | Carboplatin + capecitabine (n=1) Cisplatin + fluorouracil (n=1) Doxorubicin (n=1) Gemcitabine (n=1)  Vinorelbine + cisplatin (n=1) Vinorelbine (n=1) |
|  | Hormone therapy | Goserelin + bicalutamide (n=1) |
|  | Immunotherapy | Nivolumab (n=1) |
|  | Targeted therapy | Crizotinib (n=1) Erlotinib (n=1) Gedatolisib + palbociclib (n=1) |
| Breast | Chemotherapy | Capecitabine (n=2) Doxorubicin (n=1)  Gemcitabine + paclitaxel (n=1) Paclitaxel (n=1) |
|  | Chemotherapy + hormone therapy | Carboplatin + paclitaxel + anastrozole (n=1) |
|  | Chemotherapy + targeted therapy | Cisplatin + veliparib (n=1) |
|  | Hormone therapy | Tamoxifen (n=1) |
|  | Hormone therapy + targeted therapy | Fulvestrant + palbociclib (n=2)  Exemestane + everolimus (n=1) |
| Thyroid | Chemotherapy | Carboplatin + paclitaxel (n=2) Cisplatin (n=1) |
|  | Immunotherapy + targeted therapy | Pembrolizumab + lenvatinib (n=1) |
|  | Targeted therapy | Lenvatinib (n=2)  Lenvatinib (n=1)  Pazopanib (n=1)  Sorafenib (n=1) |
| CRC | Chemotherapy | Oxaliplatin + capecitabine (n=1) Tas 102 (n=1) |
|  | Chemotherapy + monoclonal antibody | Oxaliplatin + fluorouracil + panitumumab (n=2) Fluorouracil + irinotecan + bevacizumab (n=1)  Irinotecan + cetuximab (n=1) Irinotecan + fluorouracil + bevacizumab (n=1) Irinotecan + fluorouracil + cetuximab (n=1)  Bevacizumab + fluorouracil (n=1) |
| Pancreatic | Chemotherapy | Fluorouracil (n=1)  Cisplatin + gemcitabine (n=1)  Fluorouracil + irinotecan + oxaliplatin (n=1) |
| Neuroendocrine | Chemotherapy | Capecitabine + temozolomide (n=1)  Carboplatin + etoposide (n=1)  Cisplatin + etoposide (n=1)  Folinic acid + fluorouracil + irinotecan (n=1)  Folinic acid + fluorouracil + oxaliplatin (n=1)  Irinotecan (n=1) |
| Gynaecologic | Chemotherapy | Paclitaxel + carboplatin (n=2)  Carboplatin + pegylated liposomal doxorubicin + gemcitabine (n=1) |
|  | Immunotherapy | Avelumab (n=1) |
| Cholangiocarcinoma | Chemotherapy | Capecitabine (n=1)  Cisplatin + gemcitabine (n=1) |
| Neuroblastoma | Chemotherapy | Topotecan + temozolomide (n=2)  Cyclophosphamide + vincristine + doxorubicin + carboplatin + etoposide (n=1) Gemcitabine (n=1) |
|  | Targeted therapy | Pazopanib (n=1) |

CRC, colorectal cancer; MASC, mammary analogue secretory carcinoma; NSCLC, non-small-cell lung cancer.

**Supplementary Figure S1. Summary of best individual responses to entrectinib and prior therapies, according to prior therapy and documented progression.** (A) Patients with documented progression on most recent prior therapy. (B) Patients with no documented progression on most recent prior therapy. (C) Patients with no prior systemic therapy. CR, complete response; CRC, colorectal cancer; GI, gastrointestinal; MASC, mammary analogue secretory carcinoma; NE, not evaluable; NSCLC, non-small-cell lung cancer; PD, progressive disease; PR, partial response; SD, stable disease.


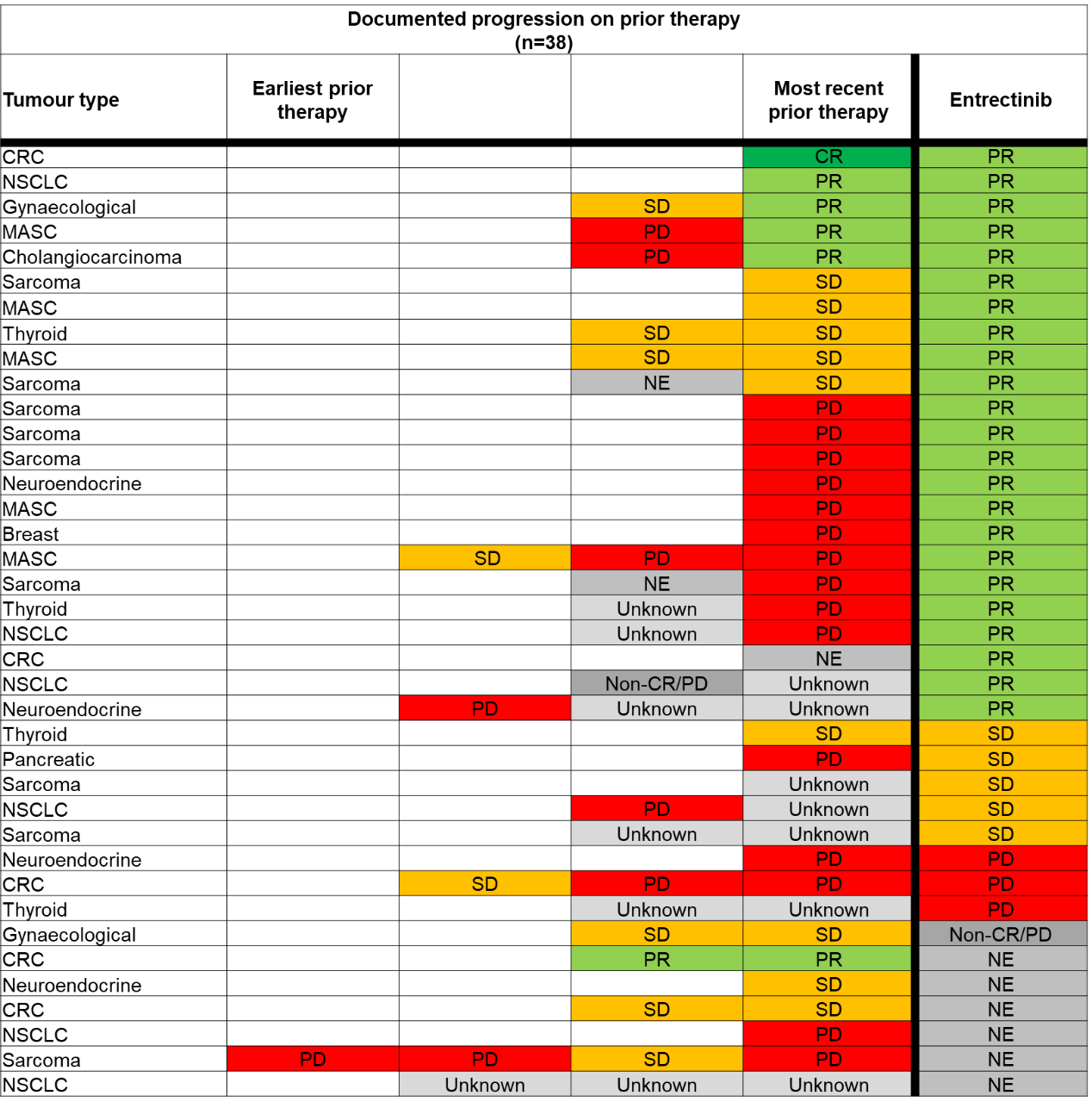
**A**

**B**

**
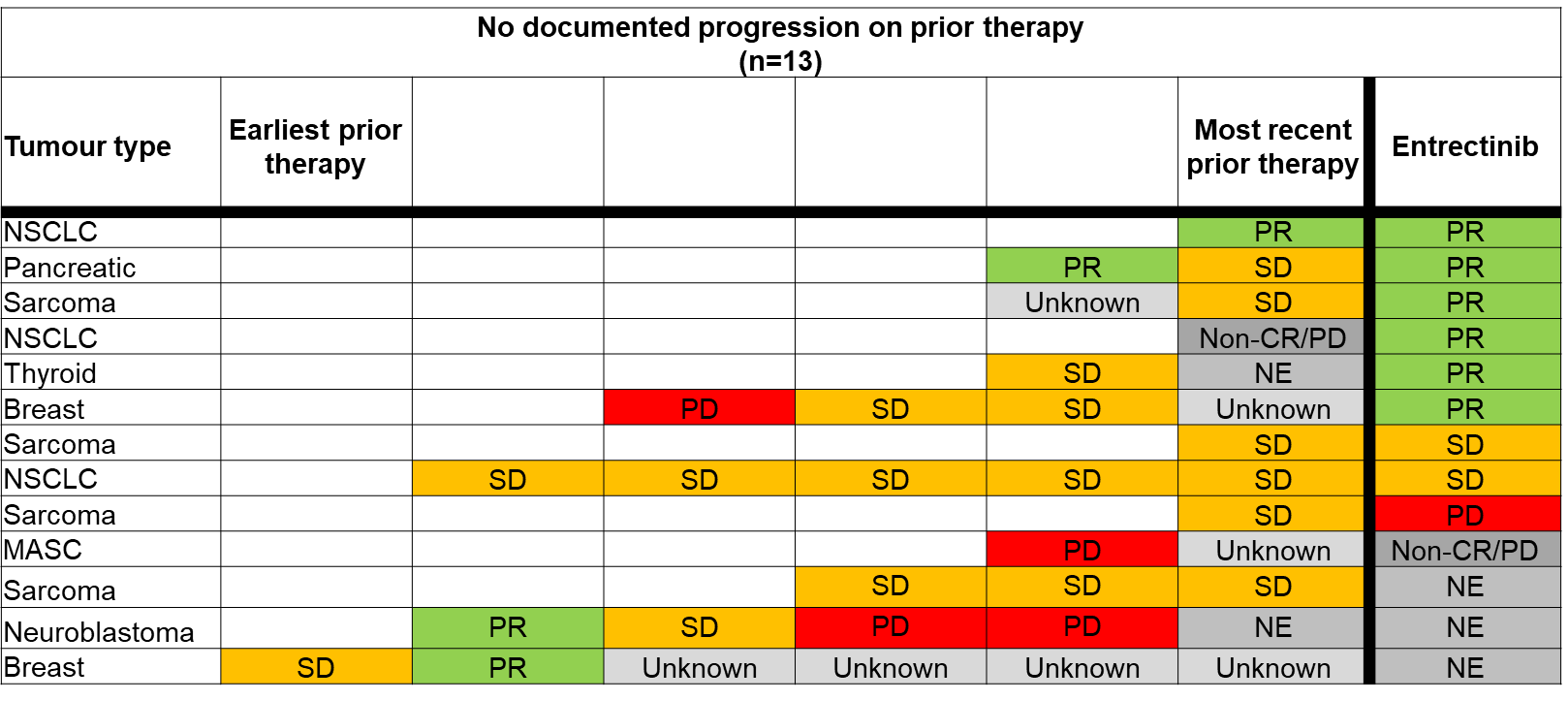
**

**
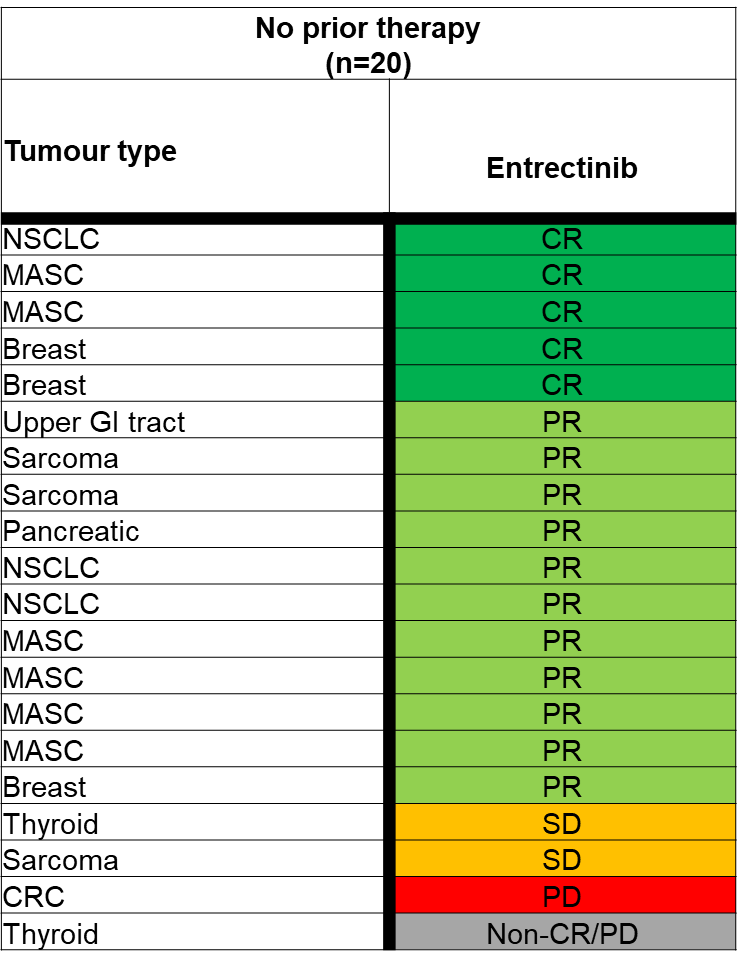
C**


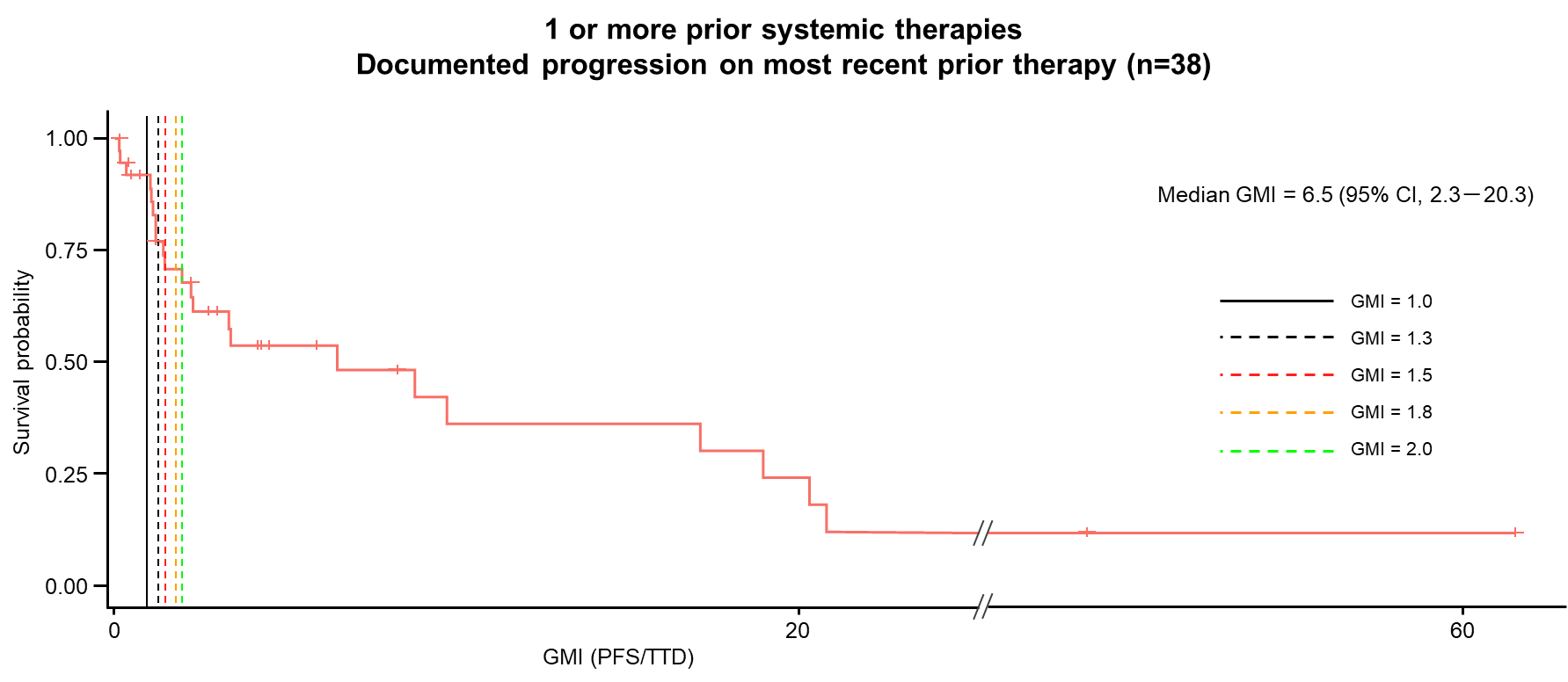
**Supplementary Figure S2. Kaplan–Meier curve of GMI in patients with documented progression on most recent prior therapy (n=38), taking censoring into account.** GMI is defined as ratio of PFS on entrectinib to time-to-discontinuation on most recent prior therapy. The dashed lines indicate a GMI ratio of 1.3 (threshold for clinically meaningful benefit; black), 1.5 (red), 1.8 (yellow) and 2.0 (green). The black line indicates a ratio of 1.0. Crosses indicate patient has been censored. CI, confidence interval; GMI, growth modulation index; PFS, progression-free survival; TTD, time-to-discontinuation.
